# Localization of GPSM2 in the Nucleus of Invasive Breast Cancer Cells Indicates a Poor Prognosis

**DOI:** 10.3389/fonc.2020.00227

**Published:** 2020-03-03

**Authors:** Mingming Deng, Zhe Zhang, Bofang Liu, Kezuo Hou, Xiaofang Che, Xiujuan Qu, Yunpeng Liu, Xuejun Hu, Ye Zhang, Qingjie Lv

**Affiliations:** ^1^Department of Respiratory and Infectious Disease of Geriatrics, The First Hospital of China Medical University, Shenyang, China; ^2^Department of Pulmonary and Critical Care Medicine, Center of Respiratory Medicine, China-Japan Friendship Hospital, Beijing, China; ^3^Graduate School of Peking Union Medical College, Chinese Academy of Medical Science and Peking Union Medical College, Beijing, China; ^4^Department of Pathology, Shengjing Hospital of China Medical University, Shenyang, China; ^5^Department of Medical Oncology, The First Hospital of China Medical University, Shenyang, China; ^6^Department of Medical Oncology, Sir Run Run Shaw Hospital, College of Medicine, Zhejiang University, Hangzhou, China; ^7^The First Laboratory of Cancer Institute, The First Hospital of China Medical University, Shenyang, China

**Keywords:** GPSM2, DYNC1I1, breast cancer, prognosis, subcellular localization

## Abstract

**Purpose:** GPSM2 (G protein signaling modulator 2) was reported to be involved in the cell division of breast cancer cells. Additionally, cytoplasmic dynein may mediate the transport process of GPSM2. DYNC1I1 (Cytoplasmic dynein 1 intermediate chain 1) is the most common cargo-binding subunit of dynein. However, the relationship between GPSM2 and DYNC1I1 and its clinical value is unclear.

**Methods:** Immunohistochemical staining was performed for assessment of GPSM2 and DYNC1I1 expression. Immunoprecipitation analysis was used to assess the interaction between GPSM2 and DYNC1I1.

**Results:** GPSM2 was correlated with clinical characteristics of breast cancer patients and is an unfavorable independent prognostic factor. In addition, nuclear expression of GPSM2 is an unfavorable independent prognostic factor (HR = 2.658, 95% CI = 1.490–4.741, *p* = 0.001). GPSM2 and DYNC1I1 are known to form a complex in breast cancer cells. Patients who were positive for expression of both DYNC1I1 and GPSM2 presented with shorter recurrence-free survival than other patients. Importantly, patients with GPSM2 nuclear expression showed higher DYNC1I1 expression.

**Conclusion:** GPSM2 was an independent prognostic factor in breast cancer and nuclear expression of GPSM2 was significantly associated with poor prognosis, which was related to the positive expression of DYNC1I1. Examination of both GPSM2 and DYNC1I1 is necessary to establish a prognosis in breast cancer patients.

## Introduction

Breast cancer is the most common malignant tumor affecting women worldwide, with ~1.38 million new-onset patients each year and is responsible for 46 million deaths to date (1). Despite advancements in chemotherapy and targeted therapy (2), a significant proportion of patients are at risk of tumor recurrence and metastasis. Therefore, in-depth study of the molecular mechanisms of breast cancer and prognosis biomarkers is essential to improve patient outcomes.

G-protein-coupled receptors (GPCRs) regulate numerous essential biological functions (3, 4). The abnormal expression of GPCRs is associated with occurrence and development of diverse types of cancer, including breast cancer (5, 6). GPSM2 (G protein signaling modulator 2) belongs to a protein family that regulates activation of G proteins (7), which then inhibit GPCR signaling by inhibiting GDP release from Gα subunits (8). GPSM2 is also widely recognized as a determinant of mitotic spindle orientation (9, 10). There are several reports of the role of GPSM2 in cancer. For example, in hepatocellular carcinoma (11) and pancreatic cancer (12), overexpression of GPSM2 promotes tumor progression and is related to prognosis. In addition, one study has observed aberrant expression of GPSM2 in breast cancer (13), but, its clinical value needs to be further explored.

Cytoplasmic dynein is a large protein complex, and has a wide range of cellular functions, including cargo transport (including vesicles, growth factors, and transcription factors) from the cytoplasm to the nucleus (14). DYNC1I1 (cytoplasmic dynein 1 intermediate chain 1) is the most common cargo binding subunit of dynein (15). The role of DYNC1I1 in cancer is controversial. One study reported a tumor-suppressive function of DYNC1I1 in GBM via transport of SK2 (16). Another study indicated, DYNC1I1 promoted gastric cancer progression by increasing NF-kB nuclear translocation (17). Moreover, several studies showed dynein could regulate mitotic spindle localization by mediation of the transport process of GPSM2 (9, 18). However, the clinical value of DYNC1I1 and the relationship between GPSM2 and DYNC1I1 in breast cancer is unclear.

In this study, we identified GPSM2 as an indicator of poor prognosis in breast cancer. Importantly, nuclear expression of GPSM2 was significantly associated with even worse prognosis. In addition, subcellular localization of GPSM2 was correlated with the expression of DYNC1I1, which also indicated a poor prognosis. In summary, examination of both GPSM2 and DYNC1I1 is necessary for accurate prognosis in breast cancer patients.

## Materials and Methods

### Patients and Tissue Samples

This study was approved by The Human Ethics Review Committee of the China Medical University. A total of 219 primary breast cancer tissue samples were obtained for the study. These 219 patients were diagnosed with invasive breast cancer and had undergone surgery at the Shengjing hospital of China medical university. None of these patients had undergone chemotherapy or radiotherapy before surgery. The clinical criteria of patient recruitment are: (1) All patients recruited had unilateral BC and were histologically diagnosed. (2) Any patient who had distant metastasis or received preoperative radiotherapy, chemotherapy, hormonal therapy, or any other anticancer therapy before surgery was excluded. (3) Patients with serious complications, such as heart disease, cerebrovascular disease, diabetes, or other malignant diseases, were excluded. (4) Complete clinicopathological data for further analysis were available. (5) All patients were followed up through medical appointments or by telephone.

About the follow-up period of the patients, the median follow-up time of patients in this study was 34 months (range 4–73). The treatment information of all patients was obtained from medical archives. As follows, among the 176 patients with stage II–III, all patients received standard adjuvant chemotherapy; a total of 124 patients with hormone receptor-positive were treated with endocrine therapies; Trastuzumab was used in 31 of 50 HER2-positive cases. Clinical characteristics of the tissue donors, such as age, sex, age at initial diagnosis, and stage at diagnosis (tumor, node status, metastasis, and TNM classification), were obtained from medical records and pathological reports.

### Immunohistochemistry (IHC) Analysis

IHC staining was performed as described previously (19). Anti-human GPSM2 rabbit antibody was used at a dilution of 1:100 (ab84571; Abcam, Cambridge, UK); Anti-human DYNC1I1 mouse antibody was used at a dilution of 1:100 (ab23905; Abcam, Cambridge, UK); phosphate-buffered saline was used as a negative control. Each section was evaluated and scored independently by two pathologists. A semi-quantitative scoring system was used in this assay. Intensity was scored as “0” (negative), “1” (weak), “2” (moderate), and “3” (strong), and the percentages of tumor cells within each category were calculated. The percentage score was multiplied by the staining intensity score to generate the IHC score. The histological score range was from zero (minimum) to 300 (maximum). Positive expression was defined as detectable immunoreactions with an IHC score > 10.

### Cell Lines and Cultures

The human breast cancer cell lines MDA-MB-231 was purchased from the Cell Bank of Type Culture Collection of Chinese Academy (Shanghai, China). MDA-MB-231 cells were grown in L15 (Invitrogen), and supplemented with 10% fetal bovine serum and maintained at 37°C with 5% CO_2_.

### Bioinformatics

The GEPIA website (20) was used to predict gene correlation in cancer. GEPIA is an online tool that provides expression analysis functions for TCGA and GTEx data.

### Western Blot (WB) and Immunoprecipitation (IP) Analyses

WB and IP analyses were performed as previously described (21). The following primary antibodies were used: GPSM2 rabbit polyclonal antibody (1:1,000; Abcam, Cambridge, UK, ab84571); DYNC1I1 mouse polyclonal antibody (1:1,000; Abcam, Cambridge, UK, ab23905); actin mouse polyclonal antibody (1:250; Santa Cruz Biotechnology, sc-47778).

### Statistical Analysis

Statistical analysis was performed using SPSS 13.0 statistical software (SPSS Inc., Chicago, IL, USA). Spearman's correlation coefficient was calculated to examine the association between continuous variables. The chi-square test was performed to analyses relationships between categorical variables. For the continuous variables, differences between three or more groups were assessed using one-way analysis of variance (ANOVA) with the *post-hoc* Tukey multiple comparison test (for normally distributed data) or Kruskal-Wallis test (for non-normal distribution). Differences between two groups were assessed using the *t*-test (normally distributed data) or Mann-Whitney test (non-normal distribution). Survival curves were analyzed by the Kaplan-Meier and log rank test. Cox's proportional hazard method was performed for multivariate analysis to identify the independent prognostic factors. *p* < 0.05 was considered significant. Relapse-free survival (RFS) was defined as the time from initial surgery to local recurrence, regional recurrence, or distant metastasis but not including disease-related death.

## Results

### The Expression of GPSM2 Correlates With Clinical Characteristics and Prognosis in Invasive Breast Cancer Patients

**Figure 1:**
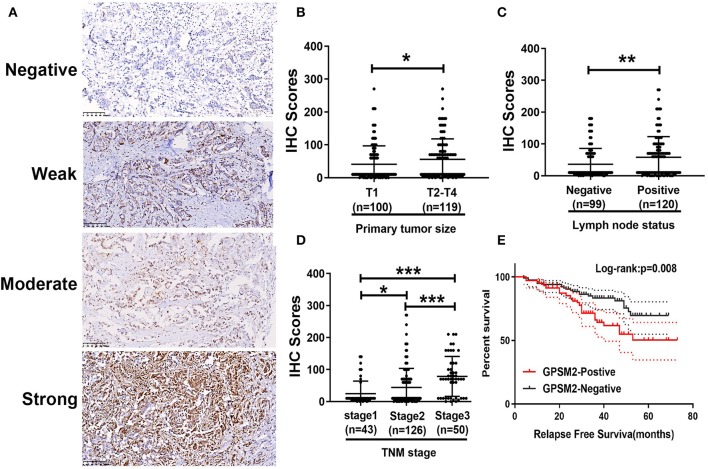
The expression of GPSM2 correlates with clinical features and indicates prognosis in breast patients. **(A)** Representative images of GPSM2 staining in human breast cancer tissue samples: “–” (negative staining), “+” (weak staining), “++” (moderate staining), and “+++” (strong staining); **(B)** GPSM2 IHC scores of samples with advanced tumor sizes were significantly increased; **(C)** GPSM2 IHC scores of samples related to positive lymph node status were significantly increased; **(D)** GPSM2 IHC staining scores were significantly increased with advanced TNM stage; **(E)** Kaplan-Meier survival analysis and log-rank test indicated that positive of GPSM2 expression was associated with poor overall survival (*p* = 0.008); *p < 0.05, **p < 0.01, and ***p < 0.001 (vs. control group).

To investigate the clinical value of GPSM2 in invasive breast cancer, we performed IHC analysis of 189 breast cancer tissue samples with available long-term follow-up medical records. Representative images of each level of staining are shown in Figure 1A. The IHC score of GPSM2 staining was significantly increased along with advanced T stage (Figure 1B), lymph node metastasis (Figure 1C) and advanced TNM stage (Figure 1D). Based on IHC scores, patients were divided into two group: GPSM2-positive and GPSM2-negative. Furthermore, a chi-square test showed that high GPSM2 expression was significantly correlated with increased T stage (*p* = 0.029), lymph node metastasis (*p* = 0.002), and higher TNM stage (*p* < 0.001) (Table 1). GPSM2-positive patients presented with a shorter relapse-free survival (RFS) time than GPSM2-negative patients (*p* = 0.008) (Figure 1E). The results demonstrated that GPSM2 could be a prognosis biomarker of invasive breast cancer patients.

**Table 1:** Correlation between GPSM2 expression and clinical characteristics in breast cancer patients (*n* = 219).

**Clinical** **Pathological**	**Number**	**GPSM2 expression**		***P*-value**
**Parameters**		**Negative (*n* = 128)**	**Positive (*n* = 91)**	
Age				0.450
<60	183	109	74	
≥60	36	19	17	
ER				0.827
Negative	103	61	42	
Positive	116	67	49	
PR				0.809
Negative	111	64	47	
Positive	108	64	44	
HER2				0.467
Negative	169	101	68	
Positive	50	27	23	
P53				0.525
Negative	97	59	38	
Positive	122	69	53	
Ki67				0.843
Negative	69	41	28	
Positive	150	87	63	
Depth of invasion				0.029^*^
T1	113	74	39	
T2–T4	106	54	52	
LN metastasis				0.002^*^
No	99	69	30	
Yes	120	59	61	
TNM stage				<0.001^*^
I	43	35	8	
II	126	78	48	
III	50	15	35	

* *Significant correlation*.

### Nucleus Localization of GPSM2 Is Significantly Correlated With Poor Clinical Outcome

**Figure 2:**
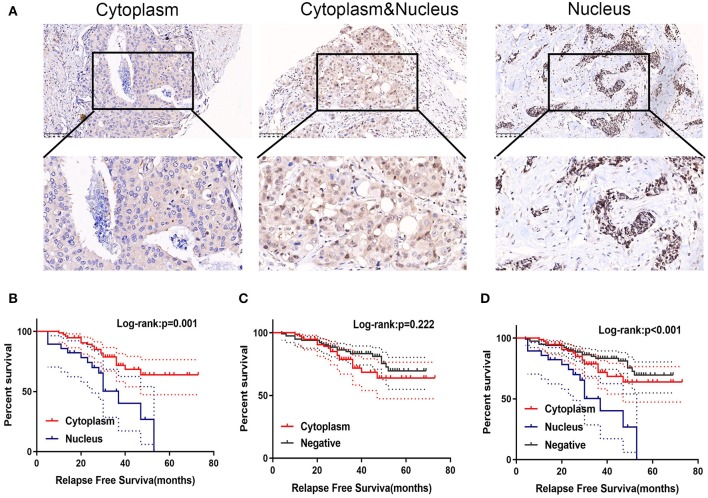
Positive of GPSM2 nucleus expression is significantly related with worse prognosis. **(A)** GPSM2 staining in different subcellular regions. **(B)** Kaplan-Meier survival analysis and log-rank test indicated that the GPSM2-nucleus patients presented with a shorter RFS time than GPSM2-cytoplasm patients (*p* = 0.001). **(C)** Kaplan-Meier survival analysis and log-rank test showed no significant difference between the RFS time of the GPSM2-cytoplasm group and GPSM2-negative group (*p* = 0.22). **(D)** Kaplan-Meier survival analysis and log-rank test demonstrated RFS of patients in the GPSM2-nucleus group was significantly reduced compared with that in other patients (*p* < 0.001).

We examined the subcellular localization of GPSM2 in breast cancer tissues. As shown in Figure 2A, the subcellular distribution of GPSM2 contained three forms: cytoplasm-positive, cytoplasm and nucleus-positive, and nucleus-positive. Comparing the staining intensity between nuclear and cytoplasmic 91 tumors were divided into two groups: 28 tumors were categorized as having high nuclear expression and 63 tumors were categorized as exhibiting high cytoplasmic expression. In Figure 2B, patients with GPSM2-nucleus localization presented with a shorter RFS time than GPSM2-cytoplasm patients (*p* = 0.001). There was no significant difference between the RFS time of the GPSM2-cytoplasm and GPSM2-negative groups (*p* = 0.222) (Figure 2C). In the total population, RFS of patients with GPSM2-nucleus was significantly reduced compared with that in other patients (*p* < 0.001) (Figure 2D). Furthermore, a chi-square test showed that compared with GPSM2-cytoplasm group, GPSM2-nucleus group was significantly correlated with HER2 receptor-positive (*p* = 0.010), Ki67-positive (*p* = 0.001), increased T stage (*p* = 0.001), lymph node metastasis (*p* = 0.015), and higher TNM stage (*p* = 0.029) (Table 2).

**Table 2:** Correlation between GPSM2 subcellular localization and clinical characteristics in breast cancer patients (*n* = 91).

**Clinical** **Pathological**	**Number**	**GPSM2 expression**		***P*–value**
**Parameters**		**Cytoplasm (*n* = 63)**	**Nucleus (*n* = 28)**	
Age				0.107
<60	74	54	20	
≥60	17	9	8	
ER				0.938
Negative	42	31	11	
Positive	49	32	17	
PR				0.263
Negative	47	35	12	
Positive	44	28	16	
HER2				0.010^*^
Negative	68	52	16	
Positive	23	11	12	
P53				0.089
Negative	38	30	8	
Positive	53	33	20	
Ki67				0.001^*^
Negative	28	23	5	
Positive	63	30	23	
Depth of invasion				0.001^*^
T1	39	34	5	
T2-T4	52	29	23	
LN metastasis				0.015^*^
No	30	25	5	
Yes	61	38	23	
TNM stage				0.029^*^
I	8	4	4	
II	48	39	9	
III	35	20	15	

* *Significant correlation*.

In addition, Cox proportional hazards model was used to analyze the impact of clinical and pathological parameters on the prognosis of patients. Univariate analysis showed that T stage (HR = 2.350, 95% confidence interval [CI] = 1.602–4.955, *P* < 0.001), N stage (HR = 2.914, 95% CI = 1.657–5.127, *P* < 0.001), TNM stage (HR = 1.730, 95% CI = 1.179–2.538, *P* = 0.005), PR status (HR = 0.525, 95% CI = 0.318–0.866, *P* = 0.012), KI67 status (HR = 2.050, 95% CI = 1.133–3.710, *P* = 0.018) and GPSM2-nucleus expression (HR = 3.902, 95% CI = 2.250–6.767, *P* < 0.001) were risk factors for prognosis. In additional, Age(HR = 0.755, 95% CI = 0.389–1.464, *P* = 0.488), ER status (HR = 0.688, 95% CI = 0.421–1.123, *P* = 0.488), HER2 status (HR = 1.026, 95% CI = 0.558–1.886, *P* = 0.935), P53 status (HR = 1.363, 95% CI = 0.830–2.241, *P* = 0.221) were not showed significant effect on prognosis. Furthermore, multivariate analysis showed that GPSM2-nucleus expression (HR = 2.658, 95% CI = 1.490–4.741, *P* = 0.001) were independent risk factors for prognosis. Meanwhile, T stage (HR = 1.529, 95% CI =0.799–2.926, *P* = 0.200), N stage (HR = 1.903, 95% CI = 0.959–3.775, *P* = 0.066), TNM stage (HR = 1.014, 95% CI = 0.634–1.620, *P* = 0.255), PR status (HR = 0.678, 95% CI = 0.397–1.158, *P* = 0.154), KI67 status (HR =1.678, 95% CI =0.916–3.073, *P* = 0.094) are not independent risk factors for prognosis (Table 3). These results indicated the prognosis value of nuclear localization of GPSM2 in breast cancer patients.

**Table 3:** Cox regression analysis of overall survival in breast cancer patients.

**Variables**	**Univariate analysis**			**Multivariate analysis**		
	**HR**	**95% CI**	***P*-value**	**HR**	**95% CI**	***P*-value**
Age (years)	0.755	0.389–1.464	0.488			
pT stage	2.350	1.602–4.955	<0.001^*^	1.529	0.799–2.926	0.200
pN stage	2.914	1.657–5.127	<0.001^*^	1.903	0.959–3.775	0.066
pTNM stage	1.730	1.179–2.538	**0.005**	1.014	0.634–1.620	0.255
ER	0.688	0.421–1.123	0.134	0.678	0.397–1.158	0.154
PR	0.525	0.318–0.866	0.012^*^	1.678	0.916–3.073	0.094
HER2	1.026	0.558–1.886	0.935			
P53	1.363	0.830–2.241	0.221			
KI67	2.050	1.133–3.710	0.018^*^			
GPSM2-nucleus expression	3.902	2.250–6.767	<0.001^*^	2.658	1.490–4.741	**0.001**
DYNC1I1 expression	3.260	1.956–5.436	<0.001^*^	1.992	1.082–3.668	0.027^*^

*Features with a p < 0.05 in univariate analysis were taken into multivariate analysis*.

* *Significant correlation*.

### GPSM2 and DYNC1I1 Form a Complex in Breast Cancer Cells

**Figure 3:**
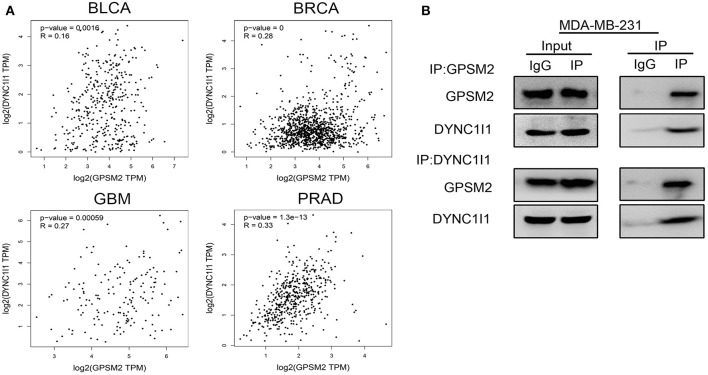
GPSM2 interacts with DYNC1I1 in breast cancer cells. **(A)** GEPIA website analysis the correlation between GPSM2 and DYNC1I1; **(B)** Immunoprecipitation assay was performed to detect the interaction between GPSM2 and DYNC1I1 in breast cancer MDA-MB-231 cells.

Next, we examined the mechanisms of GPSM2 subcellular localization. DYNC1I1 is an important cargo binding subunit of cytoplasmic dynein. So, we explored the relationship of DYNC1I1 and GPSM2. First, we used the GEPIA Website to analyze the correlation between GPSM2 and DYNC1I1. In Figure 3A, GPSM2 was positively correlated with DYNC1I1 in several cancers, such as bladder urothelial carcinoma (BLCA, *R* = 0.16, *p* = 0.0016), breast invasive carcinoma (BRCA, *R* = 0.28, *p* = 0), glioblastoma multiforme (GBM, *R* = 0.27, *p* < 0.001) and prostate adenocarcinoma (PRAD, *R* = 0.33, *p* < 0.001). Next, we performed immunoprecipitation analysis using the breast cancer cell line MDA-MB-231. As shown in Figure 3B, anti-GPSM2 antibodies efficiently immunoprecipitated DYNC1I1. Further, anti-DYNC1I1 antibodies also efficiently immunoprecipitated GPSM2. These results indicated that GPSM2 and DYNC1I1 formed a complex in breast cancer cells and may play an important role in BRCA patients.

### The Expression of DYNC1I1 Is Correlated With Clinical Characteristics and Prognosis in Invasive Breast Cancer

**Figure 4:**
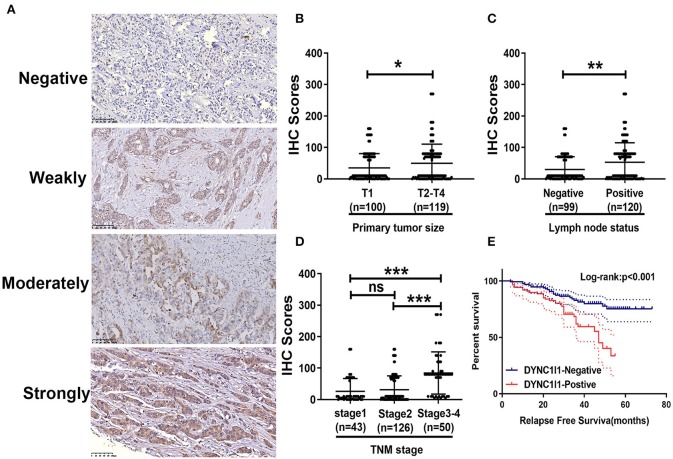
The expression of DYNC1I1 correlates with clinical characteristics and poor prognosis in breast cancer patients. **(A)** Representative images of DYNC1I1 staining in human breast cancer tissue samples: “–” (negative staining), “+” (weak staining), “++” (moderate staining), and “+++” (strong staining); **(B)** The IHC score of DYNC1I1 did not differ significantly between different tumor sizes; **(C)** DYNC1I1 IHC scores of samples related to positive lymph node status were significantly increased; **(D)** DYNC1I1 IHC staining scores were significantly increased with advanced TNM stage; **(E)** Kaplan-Meier survival analysis and log-rank test indicated that positive DYNC1I1 expression was associated with poor overall survival (*p* < 0.001); *p < 0.05, **p < 0.01, and ***p < 0.001 (vs. control group).

Next, in order to investigate the clinical value of DYNC1I1 in invasive breast cancer, we performed IHC analysis. Representative images of each level of DYNC1I1 staining are shown in Figure 4A. There were no significant differences between the expression of DYNC1I1 in different T stages (Figure 4B). Moreover, the IHC score of DYNC1I1 staining was significantly increased along with lymph node metastasis (Figure 4C) and advanced TNM stage (Figure 4D). According to IHC scores, patients were divided into two groups: DYNC1I1-positive and DYNC1I1-negative. Furthermore, a chi-square test showed that high DYNC1I1 expression was significantly correlated with ages (*p* = 0.042), PR-negative (*p* = 0.041), increased T stage (*p* < 0.001), lymph node metastasis (*p* = 0.007), and higher TNM stage (*p* < 0.001) (Table 4). Moreover, DYNC1I1-positive patients presented with a shorter RFS time than GPSM2-negative patients (*p* < 0.001) (Figure 4E). Additionally, the DYNC1I1-positive group exhibited a shorter RFS rate (HR = 1.992, 95% CI = 1.082–3.668, *p* = 0.027) (Table 3). The results demonstrated that DYNC1I1 is an independent risk factor, and may be a biomarker for the prognosis of invasive breast cancer patients.

**Table 4:** Correlation between DYNC1I1 expression and clinical characteristics in breast cancer patients (*n* = 219).

**Clinical** **Pathological**	**Number**	**DYNC1I1 expression**		***P*-value**
**Parameters**		**Negative (*n* = 131)**	**Positive (*n* = 88)**	
Age				0.042^*^
<60	183	104	79	
≥60	36	27	9	
ER				0.471
Negative	103	59	44	
Positive	116	72	44	
PR				0.041^*^
Negative	111	65	52	
Positive	108	72	36	
HER2				0.107
Negative	169	106	63	
Positive	50	25	25	
P53				0.167
Negative	97	63	34	
Positive	122	68	54	
Ki67				0.269
Negative	69	45	24	
Positive	150	86	64	
Depth of invasion				<0.001^*^
T1	113	81	32	
T2–T4	106	50	56	
LN metastasis				0.007^*^
No	99	69	30	
Yes	120	62	58	
TNM stage				<0.001^*^
I	43	32	11	
II	126	86	40	
III	50	13	37	

* *Significant correlation*.

### Correlation Between GPSM2 and DYNC1I1 and Prognosis in Invasive Breast Cancer Patients

**Figure 5:**
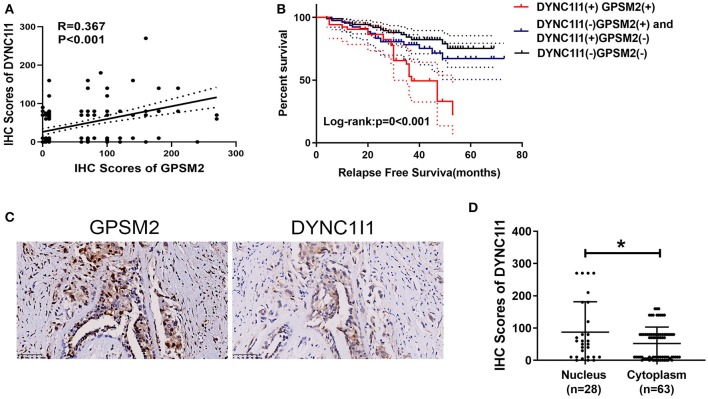
Correlations between GPSM2 expression and DYNC1I1 in breast cancer patients. **(A)** The IHC scores of GPSM2 is positively correlated with the IHC scores of DYNC1I1 (*R* = 0.367, *p* < 0.001); **(B)** Kaplan-Meier survival analysis and log-rank test indicated that the RFS of patients exhibiting expression of both DYNC1I1 and GPSM2 was significantly reduced compared to other patients (*p* < 0.001); **(C)** GPSM2 and DYNC1I1 expression; **(D)** IHC scores of DYNC1I1 in the GPSM2-nucleus group were significantly higher than the GPSM2-cytoplasm group (*p* < 0.05); *p < 0.05 (vs. control group).

Due to the clinical value of GPSM2 and DYNC1I1 in BRCA and importance of the GPSM2-DYNC1I1 complex, we aimed to explore the relationship between GPSM2 and DYNC1I1 in BRCA patients. As shown in Figure 5A, the expression of GPSM2 was positively correlated with that of DYNC1I1 (*R* = 0.367, *p* < 0.001). In addition, the RFS for patients exhibiting positive expression of both DYNC1I1 and GPSM2 was significantly reduced compared to that of the other patients (*p* < 0.001). GPSM2/DYNC1I1 expression is shown in Figure 5C. Our results showed the prognosis value of the GPSM2-DYNC1I1 complex.

### Nuclear Localization of GPSM2 Was Correlated With Positive Expression of DYNC1I1

Next, we explored the relationship between nuclear localization of GPSM2 and DYNC1I1 in invasive breast cancer. As shown in Figure 5, the IHC scores of DYNC1I1 in the GPSM2-nucleus group were higher than those of the GPSM2-cytoplasm group (*p* < 0.05). Furthermore, a chi-square test showed that nuclear localization of GPSM2 was correlated with positive expression of DYNC1I1 (Table 5).

**Table 5:** Correlations between GPSM2 and DYNC1I1 expression levels in patients with breast cancer.

**DYNC1I1 expression**	**GPSM2 expression**			***P*-values**
	**Negative**	**Cytoplasm**	**Nucleus**	
Negative	87	36	8	<0.001
Positive	41	27	20	

## Discussion

In the past three decades, the incidence of breast cancer in China has been rising, and has become the primary malignant tumor in females (22, 23). In order to increase both RFP and overall survival in breast cancer patients, investigation of drug targets and prognosis biomarkers is essential. Previous study identified an interaction of GPSM2 with G alpha proteins. Subsequent research showed that GPSM2 was involved in the regulation of asymmetric cell division and spindle orientation. Additionally, several studies indicated that GPSM2 is essential to the development of normal hearing (24). GPSM2 mutations are known to cause brain malformations and hearing loss in Chudley-McCullough syndrome (25–27). Embryonic development and tumorigenesis have similar molecular mechanisms, and thus, GPSM2 may play an important role in both.

A previous study reported that GPSM2 was overexpressed in hepatocellular carcinoma and related to poor prognosis of these patients (11). Another study in pancreatic cancer showed GPSM2 promoted the proliferation and migration ability of CD133+ pancreatic cancer stem cells (12). In addition, one study suggested GPSM2 was involved in the cell division of breast cancer cells and was regulated by PBK/TOPK (13). However, the clinical and prognosis value of GPSM2 in breast cancer has not been elucidated. Based our IHC results, we are the first to report that the expression of GPSM2 was correlated with patient prognosis and clinicopathological factors. Importantly, our results indicated GPSM2 nuclear expression was found in patients with the shortest RFS. In additional, other pathology characteristics (such as, hormone receptor negativity, HER2 status, high Ki-67 proliferation index, pT stage) did not shown affect survival in this study according the result of multivariate COX analysis. Several reasons may exist for this discrepancy, including the number of samples was relatively small, heterogenous of patients' treatments. These issues need to be addressed in future studies evaluating a larger sample population. Taken together, we report here the clinical value of GPSM2, especially GPSM2 nuclear expression, in breast cancer.

Another important finding of our study was that subcellular localization of GPSM2 was correlated with the expression of DYNC1I1, which indicated a poor prognosis in breast cancer. DYNC1I1 is the intermediate chain of cytoplasmic dynein, and is an important cargo binding subunit (15). Previous studies indicated that loss of DYNC1I1 caused split hand/split foot malformation type I (28, 29). However, the role it plays in cancer remains controversial. Depending on its transport cargo, DYNC1I1 can act as a promotion or suppression factor in tumors (16, 17). Several studies showed subcellular distribution of GPSM2 was regulated by dynein (9, 18). However, the relationship between GPSM2 and DYNC1I1 has not been fully elucidated. In our study, we demonstrated by immunoprecipitation that GPSM2 and DYNC1I1 could form a complex in breast cancer cells. Moreover, the expression of DYNC1I1 was correlated with patient prognosis and clinicopathological factors. Importantly, patients with GPSM2 nuclear expression showed higher DYNC1I1 expression. These results suggested DYNC1I1 was an indicator of poor prognosis that may function via transfer of GPSM2 into the nucleus. Examination of both GPSM2 and DYNC1I1 could help to assess patient prognosis more effectively.

Given the appreciation of their role in cancer, the importance of GPCRs for anticancer drug discovery is undisputable, although very few members have been exploited in pursuit of anticancer therapies. In this study, we have confirmed the clinical value of GPSM2.Thus, GPSM2 may be a promising target for new cancer therapy through regulating GPCRs pathway (6, 30). Due the value of GPSM2 in predicting disease recurrence, it would be better to add GPMS2 to the NGS panels like Oncotype Dx (31). Including GPSM2 to Oncotype DX gene panel may have considerable benefit in predicting disease recurrence.

However, the mechanisms of why GPSM2 nucleus-positive patients presented with poorer prognoses are unclear. We speculate that this is mainly because nuclear localization of GPSM2 is unavailable to inhibit activity of Gα protein that is expressed on the membrane, thereby enhancing the activity of the GPCR pathway and promoting tumor progression. Further studies are needed to determine whether the association between GPSM2 and DYNC1I1 is direct or indirect. We plan to further investigate these questions with additional *in vitro* and *in vivo* experiments. There are two limitations in this study. Firstly, the samples involved in this study were acquired retrospectively; secondly, the number of samples was relatively small. Thus, it needs further comprehensive and in-depth prospective study with enough samples to demonstrate that nucleus expression of GPSM2 is associated with high rate of recurrence in breast cancer patients.

In conclusion, our results strongly suggested that GPSM2 was an independent prognostic factor in breast cancer. Importantly, nuclear expression of GPSM2 was significantly associated with even worse prognoses, and was related to positive expression of DYNC1I1. We believe that assessment of the combination of GPSM2 and DYNC1I1 expression could be a promising biomarker and drug target for breast cancer.

## Data Availability Statement

The datasets generated for this study can be found in the GEPIA website (20).

## Ethics Statement

The studies involving human participants were reviewed and approved by the Research Ethics Committee of China Medical University. The patients/participants provided their written informed consent to participate in this study.

## Author Contributions

QL and YZ contributed conception and design of the study. KH, XQ, YL, and XC gave the technical support. MD and ZZ performed the experiment and statistical analysis. MD wrote the manuscript. QL provided fund supports. All authors contributed to manuscript revision, read and approved the submitted version.

### Conflict of Interest

The authors declare that the research was conducted in the absence of any commercial or financial relationships that could be construed as a potential conflict of interest.
